# Reports on the Hospitals of the United Kingdom

**Published:** 1910-04-23

**Authors:** Henry Burdett


### REPORTS

ON THE

### Hospitals of the United Kingdom.

By SIR HENRY BURDETT, K.C.B., K.C.V.O.

### XL.—DAVID LEWIS NORTHERN HOSPITAL, LIVERPOOL.

This hospital was founded in 1834 with 23 beds. A much larger building was erected in 1845 in Great Howard Street, where the present site is, and it was rebuilt in 1896-7, mainly through the generosity of the late Mr. David Lewis and his trustees, who placed a sum of £120,000 at the disposal of the governors. The site consists of about 12,000 square yards, and has its most extensive frontage to Great Howard Street, being bounded on the North by the Lancashire and Yorkshire Railway and on the South by Leeds Street. The main entrance of the administration block is situated on the West, or Great Howard Street front. It is a corridor hospital, and we published the plans and a full description of them in The Hospital of March 29, 1902, pp. 442-3. The hospital contains at present 200 beds, with an average occupancy of 173. The nursing staff consists of 14 sisters and 39 nurses and probationers, in addition to the matron, so that it is none too large for the number of beds.

### The Wards.

The wards at this hospital are well designed, having ample cubic and superficial space, and present an attractive appearance. The principal wards contain 22 beds each, which is, in our judgment, the best number for this kind of ward in a general hospital, if due regard is to be paid to ease of working and the comfort of the patients. In wards containing 30 beds and upwards it is abundantly evident that the strain upon the nursing staff is undesirably great, and that, from the patients' point of view, they are too large for reasonable comfort and the ready and easy carrying on of the daily routine. The floors are of American willow, unstained but polished, and they present a much more attractive appearance than where, as in the case of the Blackburn Infirmary, the willow has been stained, for this class of wood has a handsome surface; it does not stain well, and so presents a relatively mean as opposed to an attractive and smart appearance such as in this hospital. These floors have stood the test of 12 years' wear and tear exceedingly well. Nothing indeed could be better, and we consider, in view of this experience and that of Guy's Hospital, that American willow is a wood which should be much more largely used for the purposes of floors in the future than it has been in the past. It is relatively cheap, and it is certainly most attractive for floor purposes.

### The Fittings.

The fittings throughout the hospital are modern, but they show signs of wear and tear, due mainly to the fact that the porcelain is not of a quality sufficiently strong and good to bear the hard usage which the work of general wards entails upon all the fittings in the sculleries and lavatories of a modern hospital. We should like to impress upon hospital managers generally, and upon architects too, that second-class baths and fittings are wasteful extravagance where hospitals are concerned, as the present condition of many of these appliances in several hospitals proves to demonstration. The late Sir Douglas Galton long ago urged that no bath which was glazed inside and out would stand the hard usage extended to baths in a hospital ward. This view is proved to be sound by the experiences just quoted, and especially by the fact that at one large Infirmary already, within six months of the opening, the baths and other porcelain fittings show signs of wear and tear, which Rufford baths, for instance, that have been in use for upwards of 25 years do not exhibit a trace of. No architect is justified in scheduling second-class goods for any great general hospital, and anyone who passes such goods, in the light of the experience we now possess, is in our judgment self-condemned. We hold that every contract for the fittings of bathrooms and lavatories should contain a clause that the manufacturers bind themselves to replace any or all such appliances should they prove defective within two years of the date of their being brought into use. We conclude that the managers of this hospital had none too much money at their disposal to defray the cost of erecting a hospital, and that it was for this reason that the stone staircases and the floors, where stone is used, are made of a quality and colour which no amount of energy on the part of the management, and no outlay for upkeep, will ever cause to present a smart and cleanly appearance. We hold that stone of this class should be condemned, and not used in hospitals, for it must break the heart of any matron and any managing committee to find how impossible it is to keep this large quantity of stone in staircases and floors absolutely clean and presentable.

### The Interior Working and Management.

We spent a good deal of time in going closely into the details of the arrangement and working of the wards and some of the other departments. Everywhere we found evidence that the work of the hospital is being ably supervised, and that the nursing staff is efficient and interested in the work. Drawers and cupboards and wards, bed-linen, sculleries, ward kitchens, and everything appertaining to them are notable in this hospital by reason of a smart cleanliness which proves continuous oversight and hard work on the part of everybody concerned. The meat, bread, tea, and other articles supplied to the patients are specially good, and the arrangements of the kitchen, which is in charge of a sister, were attractive, while the stores and fittings of this unit afforded little ground for criticism but much for commendation. The impression we brought away with us was that the hospital, though not supported to the extent its merits and the work it is doing deserve, is administered with a care and thoroughness highly creditable to everybody concerned in the administration of its affairs. The matron, Miss A. C. Glover, an old London Hospital sister, does her work in a manner which reflects honour upon her *alma mater*, and the Secretary and Superintendent, Mr. Joseph Unsworth, who has held his office for many years, has a pleasant personality and is devoted to the interests of his hospital. We were glad to meet the Chairman, Mr. H. Wade Deacon, who gives a large amount of time to the affairs of this institution, and who—with the officers, when their work is judged on its merits—is entitled to much more liberal support at the hands of the citizens of Liverpool than he is receiving at the present time. There is a deficiency of nearly £6,000 shown in the last year's accounts, and we certainly hope that some generous men and women who believe in the doctrine of personal service will make it their business to cooperate with Mr. Deacon in placing the finances of this interesting hospital upon a sounder and better basis. It is a good hospital, where everything is neatly and well kept. It lacks a modern, up-to-date theatre, and we venture to hope that, in the new one which is now being constructed out of the old Tank Ward, the present half-brick partitions which shut off the two small rooms on either side of the entrance hall will be taken down and extended into the ward to a line drawn from the chimney-breast of the fireplace to the opposite wall. In this way a good anæsthetic room and a sterilising room can be provided, and a theatre of ample proportions constructed, which, providing the floor is made aseptic by being covered with a suitable material, should be a great addition to the hospital.

### The Nurses' Home.

This is a long building extending the whole depth of the site, with a central corridor which is not well lighted and not adequately ventilated. The nurses' sleeping-rooms and sitting-room are on the whole good, the latter presenting an attractive appearance. It is a grave fault, however, to have placed the w.c.s in the centre of the corridor with no adequate ventilation, for they make the atmosphere of that corridor, as we found it at the time of our visit, unhealthy and disagreeable. This is a matter which deserves the immediate attention of the Committee, for the present state of things should not be allowed to continue a moment longer than the time it will take to reconstruct and ventilate properly the places in question. This can be done by building out a tower on the hospital side of this block. Otherwise the home is large and comfortably furnished, though its plan of construction is by no means good.

We have observed in the course of our inspections of these Lancashire hospitals that there is a tendency to cut salaries too low. In our view the head of the nursing department of a hospital with 200 beds, especially where there is a private staff large enough to earn nearly £1,200 each year, should not receive a less salary than £150 annually, which is the current rate to pay for such a post.

### Finance.

We should be glad to see the accounts of this hospital kept upon the Uniform System. As they are made out at present it is not possible to compare the working expenses of this hospital with those of other similar institutions. That is a great loss to a voluntary hospital like the David Lewis Northern Hospital, which has to fight hard for its existence and where the funds are none too abundant. During the year 1908, so far as we can make out, the income from all sources about met the expenditure. Seeing, however, that the year was commenced with a deficiency of nearly £6,000, and that the deficiency remains unliquidated, it seems to us essential that active steps should be taken to raise a fund to liquidate this debt, and at the same time to increase the annual subscriptions and annual donations. We are not usually in favour of hospital dinners, but it seems to us that in the present case, if Mr. Wade Deacon and his Committee were to determine to organise such a banquet, and drive home the claims of this hospital and the work it is doing for the citizens of Liverpool, the result should be to attract a number of new friends and contributors, and at the same time to raise a sum of £10,000, which would put the hospital in funds and enable its Committee to do many small things which require doing, but are at present delayed for want of money.

---

The **Hospital of St. Cross, Rugby,** and the **Shepton Mallet District Hospital** will be reported on next week.

